# Nursing in oncology ward with intertwined roles: a focused ethnography

**DOI:** 10.1186/s12912-023-01250-8

**Published:** 2023-03-24

**Authors:** Hadiseh Monadi Ziarat, Naima Seyedfatemi, Marjan Mardani-Hamooleh, Mansoureh Ashghali Farahani, AbouAli Vedadhir

**Affiliations:** 1 Department of Nursing, Nursing and Midwifery Care Research Center, Iran University of Medical Sciences, Tehran, Iran; 2 Department of Anthropology, University of Tehran, Tehran, Iran; 3 Department of Psychiatric Nursing, Iran University of Medical Sciences, Zafar Str, Vanak Sq, PO Box 1419733171, Tehran, Iran

**Keywords:** Ethnography, Nurse, Nursing care, Oncology

## Abstract

**Background:**

Characteristics of nursing care in the oncology ward depend on this ward’s specific context. This study aimed to investigate the nursing care in the oncology ward regarding the culture of this ward.

**Methods:**

This qualitative study was conducted in an oncology ward using a focused ethnographic approach. The whole nursing team of the selected ward (N = 16) participated in the study through purposeful sampling. Three methods of observation, interview, and field documents were used for data collection. Data were analyzed by Spradley’s (1980) ethnographic method.

**Results:**

‘Nursing in the oncology ward with intertwined roles’ emerged as the main theme. This theme included the following subthemes: ‘Robin Hood nurse,’ ‘a secretive nurse,’ ‘a negligent nurse,’ ‘a snitching nurse,’ ‘a complaining nurse,’ ‘an apathetic senior nurse,’ ‘a stigmatized training nurse,’ ‘a brazen-bodied nurse,’ ‘a compassionate nurse,’ ‘a moonlighting nurse,’ and ‘a drug bartender.’

**Conclusion:**

This study provided a deep cultural insight into nursing care in the oncology ward, considering the particular culture of this ward and emphasizing the nurses’ intertwined roles. These roles are on a spectrum, with positive roles, such as compassion, on one side and negative roles, such as negligence, on the other. The results of this study can be provided to nursing managers; therefore, by being aware of nurses’ roles considering the specific subculture of the oncology ward, they can provide psychological interventions to improve the mental health of reluctant and complaining nurses and ethics-based training for secretive, negligent, and snitching nurses to provide quality care to the patient.

## Background

In 2020, 19.3 million people were diagnosed with cancer, and it is estimated to reach 28.9 million in 2040 [1]. Cancer prevalence will increase in Iran by 2040, almost twice the global rate [2]. A cancer diagnosis can cause a crisis in one’s life. During the treatment period, cancer patients may experience severe physical and mental distress [3].The disease complications usually occur simultaneously, known as “a symptom cluster.” These complications consist of physical symptoms and mental distress (emotional, behavioral, and verbal indicators), including excessive fatigue, denial, fear, inner turmoil, isolation, anorexia, identity conflict, anger, anxiety, depression, and changes in physical appearance [4]. These complications bring about changes in cancer patients’ quality of life, making them vulnerable [5]. On the other hand, most of these patients have an unpleasant hospital experience [6] since their treatment period is complicated, painful, and stressful [7]. Consequently, according to the mentioned factors, the care for cancer patients in oncology wards requires special attention [8].

Nurses play a fundamental role as supporters and caregivers of patients in oncology wards since they are in contact with these patients for a long period of time and play an essential role in meeting their need for proper care [7]. As cancer is becoming more prevalent worldwide, the challenges of caring for these patients are also increasing since the care for and communication with cancer patients are highly stressful due to the lethal nature of most cancers, their complex treatment, demanding decision-making, relationship with death, and nurses’ sense of failure and emptiness when cancer patients are not cured [9]. Oncology nurses bear a considerable emotional burden [10]. They assume caring for cancer patients is futile and describe it as walking on a treadmill [11].Caring for cancer patients is physically and mentally debilitating for nurses since they witness the pain and suffering of patients and their families, exposing them to tension. Moreover, in oncology wards, increasing dejection due to caring for patients leads to compassion fatigue (physical and mental distress and fatigue caused by care) and increased burnout in nurses [8], which will negatively affect caring behavior and patient satisfaction [12]. However, if nurses perform their care behaviors efficiently in oncology wards, patients’ satisfaction, rehabilitation, and well-being will improve [3]. Therefore, nurses’ caring behavior in the oncology ward is of particular importance. Nursing care for cancer patients is highly stressful due to complex treatment, difficult decision-making, and feelings of emptiness when cancer patients are not treated [9]. Nurses believe caring for cancer patients is futile and describe it as walking on a treadmill [11]. However, providing efficient care by nurses in the oncology ward will increase patient satisfaction [3].

Several studies have addressed nursing care in the oncology ward. The main theme of a study in Iran was ‘Being a canopy for cancer patients.’ In this study, nurses provided compassionate care and emotional support, established friendly and empathetic relationships with patients, and met their needs [13]. According to research results in Germany, the themes of caring for cancer patients from the perspective of the treatment team included psychological care, cooperation of care providers, patient-caregiver relationships, and coordination and organization in care [14]. A study in Turkey showed that nurses described care for cancer patients as prioritizing patients’ requests and availability, comforting them, treating them as close family members, explaining, listening, giving hope, and having a sense of humor [15]. A study in Japan states that nurses are required to be skilled in managing their emotions while caring for cancer patients and their families [10].

According to the literature review, nursing care in the oncology ward is of great importance and has context-dependent characteristics. Since the ethnographic study emphasizes the distinct cultural context in a specific environment, observing, describing, and understanding the effect of culture on individuals’ behavior, care provided by nurses in the oncology ward can vary according to the specific subculture of this ward.

During the experience of caring for cancer patients, the researcher realized that nurses had different caring characteristics, for instance, the care accompanied by compassion or negligence and inattention to patients’ and their companions’ psychological issues. To the best of our knowledge, no study has been conducted in Iran on nursing care in the oncology ward using an ethnographic approach. Therefore, it seemed necessary to explore nursing care in this ward, considering its culture. This study aimed to investigate the nursing care in the oncology ward regarding the culture of this ward.

## Methods

### Study design

This focused ethnographic study was conducted based on Spradley’s (1980) developmental research sequence (DRS). The main purpose of this approach is to describe the cultural pattern and interpret it [16]. In focused ethnography, particular contextual characteristics of a specific group within a culture are described as they occur in everyday life [17]. Since this study focused on nursing care considering the oncology ward culture, this approach was used.

This study was conducted from December 2020 to June 2022 in one of the oncology wards of a public hospital in Tehran, the capital of Iran. This oncology referral center has eight wards, one emergency room, and four clinics. In the selection of the study setting, simplicity (single environment instead of multiple), the accessibility of the intended setting, convenience of entering the setting to collect data, and permission to enter and perform repeatable periodic activities were considered [16]. In the intended ward, patients of different ethnicities and diagnosed with various cancer types were admitted. Moreover, the nurses working in this ward were diverse in terms of age, gender, work experience, and recruitment method. In addition to nurses and nurse assistants in the ward, there were patients, physicians, secretaries, medical and nursing professors, students, and staff.

### Participants

The participants were selected considering maximum diversity in terms of age, gender, work experience, and educational qualification. The sampling was performed through the purposeful method. Considering the fact that in an ethnographic study, all individuals play an active role [18], the whole nursing team of the selected ward was considered participants (N = 16), of whom 12 were nursing graduates, and 4 were nursing assistants. In this study, 16 individuals from the nursing team participated. Of these, 12 were nursing experts, and 4 were nursing assistants. The majority of them were female (68.8%), and their mean age was 32.13 ± 5.9 years. Their ages ranged between 24 and 40 years, and their average work experience was 6.9 ± 5.07.

### Data collection

The data collection method in this study was based on the triangulation approach to obtain accurate, rich, and unbiased data, which included three methods: observation, interviews, and field documents. Participant observation was the main method for collecting the data. A total of 207 h of observation was performed in the oncology ward during the morning, evening, long-day, and night shifts on different occasions on all workdays and holidays. The researcher used the participant observation method in this study to prevent participation in the care process from disrupting patient care and to provide the opportunity for prolonged social interaction between the researcher and the ward nursing team. The researcher attended the ward with two goals: observing nurses’ practices and behaviors as well as the physical structure of the ward and participating in nursing practices appropriate to the situation. During the observations and with a broad vision and without prejudice, the researcher made an effort to consider both goals and become aware of all aspects of the physical structure of the ward and nurses and their practices, emphasizing their roles. Initially, the researcher entered the study setting as an external member and had the sense of a stranger in an unfamiliar environment. However, over time, she tried to establish a friendly relationship with the personnel and perform ward activities as an internal member to achieve the general purpose of the study, which was to investigate nursing care in the oncology ward considering its culture. The researcher took measures in this regard; for instance, during tea time, she participated in the personnel’s talks and confirmed their conversations to encourage them to continue talking. Besides, she accompanied and helped the nurses while doing the activities. For instance, when administering medications, she often accompanied them to the patient’s room and helped them dissolve the medicine. Over time, the trust and intimacy between the researcher and the staff increased. Gradually, attending the ward became a routine activity for the researcher, and when she entered the ward, everyone recognized her and considered her a member of the oncology ward. The researcher attempted to integrate the internal and external member’s points of view as the study progressed. In other words, she considered herself a nursing team member and maintained her perspective as the researcher. The researcher’s participation and observations during attendance in the ward varied according to the time, conditions, and cooperation of the personnel. The researcher gradually established nurses’ roles, accompanied them in all their practices, cooperated under their supervision if necessary, and used all three observation methods cyclically according to the research process. Considering that the main component of ethnographic research is question-observation, descriptive, structural, and comparative questions were first proposed; afterward, observations were made based on them. The combination of these three methods of observation and the resulting outcomes helped the research team to obtain accurate and rich data. Initially, descriptive questions were asked, and descriptive observations were made based on these questions in two ways: full-round and short-round observations. At the beginning of the researcher’s attendance in the ward, descriptive observations were made as full-round; however, over time, the researcher made short-round observations based on the full-round observations. An example of full-round observation questions includes “What are ward nursing team’s education, work experience, age, and gender?” and “What are ward nurses’ most frequent professional practices?” An example of short-round observation questions include, “What events and activities do nurses participate in?” and “How is the process of performing each activity?” The purpose of the descriptive observations was to collect general data about nursing culture in the oncology ward. After descriptive observations, structural questions were designed according to the main focus of the study, and focused observations were conducted accordingly to obtain more accurate data. Structural questions included “What is the medication administration process in the ward?” and “How are vital signs controlled in the ward?” The purpose of conducting such observation was to ponder the types of nurses’ roles according to the culture of the oncology ward. Gradually, the researcher made selective observations while attending the ward and cooperating in the nurses’ care activities without disturbing their practices. The purpose of this observation was to discover the conflicting dimensions of the nurses’ different roles with regard to the nursing culture in the oncology ward. This type of observation was conducted based on comparative questions such as “How is the responsibility of Robin Hood’s nurse different from that of the complaining nurse?” or “What is the difference between a drug bartender nurse and a stigmatized training nurse in terms of self-sacrifice?” Observations were recorded as field notes immediately after they ended or at the earliest opportunity. To enrich the data and complete the observations, the researcher implemented interviews informally with the nursing team. During the interviews, the researcher tried to encourage nurses to continue talking by listening to them carefully and respectfully, allowing them to give meaning to the conditions and activities in their own language and answering them in a non-judgmental style. The interview process was not predetermined and was implemented immediately after the events and interactions observed to obtain implicit and explicit meanings of the conditions and activities from the perspective of the personnel. The interview questions were based on the participants’ observed actions. The researcher conducted several informal interviews with each staff according to the situation and conditions that emerged. Eighty informal interviews were conducted with 16 nursing team members, lasting from two to twenty minutes. The informal interviews were conducted in a place close to the nurses’ caring behaviors and convenient for the participants, often in the staff resting room whose entrance was located in the nursing station, and they agreed on it. During the interviews, the researcher tried to listen prudently to the interviewees and transcribe the interviews carefully, at the earliest opportunity, using the words used by the nurses and other personnel to accurately understand what the nursing team knows. In fact, the nursing team had the role of a teacher for the researcher in identifying the nursing care culture and helped her understand the ward events and their implicit meanings. In addition to observation and interviews, field documents were also used. These documents included the form describing oncology nurses’ specialized duties, the policy of chemotherapy drugs’ storage and maintenance, the ward’s rules and regulations, and the patient’s rights charter. The researcher recorded the field notes resulting from the three methods of observations, informal interviews, and documents first in the research field and kept a concise record of them, which included unrelated phrases and sentences, in her mobile phone’s notes app. She recorded those reports during or immediately after the observation. Afterward, she recorded expanded and detailed notes based on those concise reports at the earliest opportunity at the end of the shift. All field notes, condensed and expanded, were reviewed by four participants, modified if needed, and finally approved. It should be noted that the researcher adhered to the principles stated by Spradley, namely the principle of identifying the language, the principle of verbatim, and the principle of accurate recording of facts in recording observations, informal interviews, and documents [16].

### Data analysis

This study is a part of the doctoral thesis conducted through a focused ethnographic approach and adapted from nursing Spradley’s DRS method [16]. This twelve-step method includes being in a social situation, participatory observation, recording ethnographic data, descriptive observations, domain analysis, focused observations, taxonomic analysis, selective observations, conducting component analysis, discovering cultural content, preparing a list of characteristics, and ethnographic culture and writing were analyzed [16]. In this method, for data analysis, observations, interview transcripts, and field documents were recorded by the first author as field notes and revised several times by research team members. Afterward, primary concepts were extracted through word-by-word reading and an overall consideration of the data. To this end, each field note with primary concepts obtained in a single work shift was sent to research team members via email, and they returned their comments after reviewing them. Then, every 14 days, a meeting was held in person or virtually, and a consensus was reached regarding primary concepts. Then, primary concepts obtained were analyzed in the four interconnected steps of the DRS method, including domain analysis, taxonomic analysis, componential analysis, and the discovering cultural theme, using the interactive-inductive method to identify sub-themes and the main theme. To this end, according to the nurses’ caring behavior in the oncology ward in the domain analysis stage, the research team moved from merely observing the social situation to discovering the cultural scenes and domain. The cultural domain is a category with cultural meaning that includes other smaller categories. Each domain consists of three main elements: the cover term, the included terms, and the semantic relation. The cover term is the cultural domain. The included terms are all smaller categories or interconnected primary concepts within the domain. The semantic relationship, which is the third element in all cultural domains, connects two categories. In this step, the researcher first examined all the primary concepts to identify the primary concepts corresponding to the semantic relation of the mandatory existence: ‘X is a type of Y.’ At this stage, we looked for a response to questions such as “Which primary concepts can be a type of something?” The purpose of this step was to comprehensively and accurately identify the cultural domains. Ultimately, the domain of “types of nurse roles in the oncology ward” was identified as the main theme. In the taxonomy analysis stage, the research team investigated the similarities between primary concepts within the domain of nurse roles and sought to identify sub-themes and better organize the data, which were divided into 11 subthemes of types of nurse roles in the oncology ward. In the component analysis step, the research team identified the differences and dimensions of conflict between the identified sub-themes to ensure the classification method and the identified sub-themes. Finally, in the ‘discovering the cultural theme’ step, the general semantic relationships were established, and the cultural themes were listed; consequently, the research team came to the conclusion that nurses take diverse roles in the oncology ward according to the culture of this ward and the time and place requirements, which are on two sides of the same spectrum, with positive roles on one side and negative roles on the other. It should be noted that the researcher moved back and forth between ideas, data collection, data analysis, and results.

### Trustworthiness

For the study rigor, Guba & Lincoln’s criteria, including credibility, dependability, confirmability, and transferability, and Morse’s recommendations for ethnographic studies were used [19, 20]. To establish credibility, prolonged engagement, persistent observation, rich and comprehensive descriptions, member-check, triangulation, and reflexivity were used. In order to achieve dependability, all research steps were transcribed in detail to enable other researchers to follow the results. Three nursing professors unrelated to the study were requested to review the research process, data, results, and interpretations. Transferability was ensured through the precise expression of the study method. For confirmability and providing the possibility of the research audit, all stages of the study, including data collection and analysis, and the formation of subthemes and the main theme, were explained. The research team confirmed the accuracy of the study.

### Ethical considerations

Ethical considerations included obtaining written informed consent for data collection, including observations, documenting and recording informal interviews, maintaining the principle of anonymity and confidentiality of participants’ names and related information, storing data in an encrypted computer, and the participants’ freedom to withdraw from the study at any stage of the study. Moreover, the researcher did not comment or intervene in nurses’ caring behaviors to prevent their behavioral changes, participated in the ward activities to the extent that no disruption occurred in the care provision, and made an effort to report the whole thing she observed according to the purpose of the study and without personal judgment and bias.

## Results

In this study, 16 individuals from the nursing team participated. (Table 1)

**Table 1:** Participants’ demographic characteristics

Organization position	gender	work experience (years)	employment status (years),	work shift type	marital status	age (years)
Head nurse	Female	16	Permanent	Morning	Married	40
Substitute head nurse	Female	10	Permanent	Long day	Married	35
Nurse	Male	2	Temporary-to-permanent	Rotating	Single	27
Nurse	Female	6	Corporate	Night	Married	36
Nurse	Female	6	Corporate	Night	Married	31
Nurse	Female	1	Training	Rotating	Married	25
Nurse	Female	1	Training	Rotating	Married	26
Nurse	Female	1	Training	Rotating	Married	36
Nurse	Female	1	Training	Rotating	Single	25
Nurse	Female	1	Training	Rotating	Single	25
Nurse	Female	1	Training	Rotating	Single	24
practical nurses	Male	11	Contractual	Rotating	Married	38
practical nurses	Female	7	Contractual	Rotating	Married	30
practical nurses	Male	10	Contractual	Long day	Married	42
practical nurses	Male	5	Contractual	Rotating	Single	36
practical nurses	Male	9	Contractual	Rotating	Married	44

The findings of this study were obtained using the twelve-step DRS analysis method, which was fully described. ‘Nursing in the oncology ward with intertwined roles’ emerged as the main theme. This theme had the following subthemes: ‘Robin Hood nurse,’ ‘a secretive nurse,’ ‘a negligent nurse,’ ‘a snitching nurse,’ ‘a complaining nurse,’ ‘an apathetic senior nurse,’ ‘a stigmatized training nurse,’ ‘a brazen-bodied nurse,’ ‘a compassionate nurse,’ ‘a moonlighting nurse,’ and ‘a drug bartender.’ (Table 2).

**Table 2:** The main theme, subthemes, and a sample of primary concepts

Primary concepts	Subthemes	Main theme
-Using a patient’s additional chemotherapy drugs for other patients without obtaining his/her permission -Using excess chemotherapy drugs for patients with financial problems	Robin Hood nurse	Types of nurses’ roles
- Concealing the diagnosis of the disease from the patient at the family’s request - Secrecy about the needlestick to other personnel	A secretive nurse	
- Incompletely drawing chemotherapy drugs and discarding the remaining drug inside the vial - Visual and remote monitoring of vital signs - Inattention to patients’ and companions’ requests and complaints	A negligent nurse	
- Disclosing a colleague’s error to other personnel in his/her absence - The use of the term “a snitch” for some nurses by the staff - Undermining nursing colleagues and transferring them to other wards	A snitching nurse	
- Constant complaints about their temporary transfer - Dissatisfaction with multiple shifts and high workload - Dissatisfaction with the low perks of the nursing profession	A complaining nurse	
- Finding the nursing profession boring - Nurses’ burnout over time - Continuing studies and going on an educational mission to evade the profession	An apathetic senior nurse	
-A training nurse, a subordinate - No permission to speak or object - Using the title “a training nurse” for stigmatization	A stigmatized training nurse	
- The nurse’s belief that she did not get infected with COVID-19 - The nurse’s assurance that she/he will not be reinfected with COVID-19 - Nurses sitting close to each other in the nursing station during the COVID − 19 pandemic	A brazen-bodied nurse	
- Friendly and close relationship with patients - Attention, precise care, and constant presence at the patient’s bedside - Being well-known for kindness among patients, companions, and staff	A compassionate nurse	
- Using the term “moonlighting” for some nurses by other ward personnel - Simultaneous employment of a nurse in another hospital’s COVID-19 ward or ICU	A moonlighting nurse	
- Patients’ frequent requests from the nurse to administer narcotic pain medication - The nurse’s sense of being a drug bartender	A drug bartender	

### Robin Hood nurse

Nurses used patients’ additional chemotherapy drugs for patients who lacked that drug without permission. In Iran, due to the sanctions on medicines, difficult access to chemotherapy drugs, and poor economic status of some patients who cannot afford to purchase drugs, such behavior evokes the nurse’s role as Robin Hood, although it is against their professional duties. One of the nurses said: “Most chemotherapy drugs are similar for patients, and if there is a shortage of drugs, we use one patient’s drugs for another.” Another nurse stated: “When the patient doesn’t have drugs, we take it from the patient who has more than the necessary dose.”

### A secretive nurse

Nurses secretively performed some activities, such as concealing the diagnosis of the disease from the patient at their family’s request or vice versa, concealing errors from other colleagues, and concealing the nursing managers’ performance from other nurses. One of the nurses told the other patients about one of the patients: “The patient doesn’t know that he has cancer; his family asked us not to tell him, be careful not to say anything.” One of the nurses advised the researcher to conceal errors and said: “Always remember, even if you slip up while working, don’t tell anyone in this ward; solve it yourself.” Besides, the researcher observed that the nursing managers shared some items donated to the ward among themselves without informing other staff about them.

### A negligent nurse

Negligence was visible in various dimensions of nurses’ duties as negligence in the drug therapy process, delegating tasks to the nurse assistant, the patient, or their companions, and visual examination of vital signs. The researcher witnessed the inattentiveness of the nurse when she was preparing the chemotherapy drugs; she drew the drug vial into the syringe carelessly and incompletely and discarded the remaining drug. The researcher observed that non-injectable drugs were often administered by companions or were forgotten. For instance, the nurse said to the patient’s companion: “Take these four pills and give your patient one pill every six hours.” The researcher asked the nurse: “According to the prescription, the patient should take one pill now.” The nurse said: “That’s ok, he will need it for the next hours; you know, it’s better to give him everything he needs because sometimes night-shift co-workers don’t give the drug.” One of the nurses described the reason for forgetting to perform their duties: “There is a lot to do, but there’s not enough staff; there are usually two nurses to take care of 26 patients; we forget or miss something, so we have to stay here after the shift and do them.” Nurses monitored patients’ vital signs visually. The researcher observed that nurses did not control blood pressure and pulse rate before administration of some drugs requiring them. When she asked about that, one of the nurses said: “No need to check them; we visually examine the patient’s general condition.” Moreover, some nurses delegated their duties, such as painkiller administration, to nurse assistants.

### A snitching nurse

Snitching was one of the prominent characteristics of some nurses, and other personnel recognized them as snitching nurses. This unethical role was manifested as exaggerating the errors made by colleagues, denouncing their colleagues to managers and other colleagues, and attempting to undermine their colleague’s position in the ward to transfer them to another ward. The researcher observed that some nurses met the supervisor at the end of the shift after their colleague left the ward and used expressions such as: “Why does this new staff make decisions? We weren’t allowed to talk when we were newly employed. Don’t let her talk so much. She even doesn’t do her job well. If she gives ideas too much, transfer her to the COVID-19 ward.” One of the nurses told her colleagues about another nurse: “She doesn’t do anything right at all; she just wants to evade the work; the previous shift, she wanted to inject one patient’s chemotherapy drug with another’s serum.”

### A complaining nurse

Nurses’ constant complaints were related to working conditions such as work schedule, multiple shifts, transfer to another ward, high workload, and low perks. One of the nurses said: “Recently, the work schedule has changed; 5 nurses work in day shift, but only one on the night shift. What should the unfortunate nurse with fixed night shifts do? This is really annoying; it’s hard to work here; when you look at the patients, you feel depressed”. Another nurse constantly complained about her temporary transfer to another ward and said: “I don’t do anything in the ward; I want them to understand. They don’t have the right to transfer me whenever they want”. One of the nurses was dissatisfied with the low perks: “Why do we do all the patient’s work, but there are no perks.”

### An apathetic senior nurse

Senior nurses with higher working experience mentioned fatigue, burnout, and the low value of their profession. They were less motivated to fulfill their professional duties and were devising a way to escape their profession or professional duties. The researcher observed that the senior nurse had a tendency to delegate her duties to others. She assigned the administration of non-injectable drugs to the patient’s companion and medication administration, taking ECG and checking blood sugar to nurse assistants, which was contrary to the description of their duties. One of the nurses said: “Nursing is a field in which you get worn out and tired over time. I want to get a master’s degree in another field so that I can go on an educational mission and get free.” Another senior nurse was tired of doing patients’ non-clinical tasks. She said: “That’s not the nurse’s business; why do I have to write the patient’s diet? You know, a nutritionist should check it and ask about their diet. Why do we have to do unimportant tasks when we should give care?”

### A stigmatized training nurse

Providing care for newly trained nurses was challenging. A nurse entitled ’a training nurse’ in the ward had to accept being a subordinate, remaining silent in the face of injustice, tolerating other personnel’s indifference and sometimes harsh behavior, and being content with less than their rights. Perhaps personnel’s attitude towards training nurses was due to similar experiences during their training course. One of the nurses said: “A training nurse shouldn’t object and should accept whatever others say.” Another nurse stated: “The training nurses talk too much; we had no right to object.” Newly-graduated nurses complained about their position in the ward. One of them said: “We have no right to object. They’ll say we are training nurses if we have any objection!”

### A brazen-bodied nurse

Some nurses considered themselves and their colleagues as brazen-bodied Esfandiar, a legendary hero in Ferdowsi’s Shahnameh^1^. They believed no risk threatened their physical and mental health, and they expected a strong and invulnerable body from themselves and their colleagues. The researcher observed that although there were two patients with COVID-19 in the inpatient ward, the personnel did not follow the personal protection principles properly. Despite enough space in the nursing station, they were sitting very close to each other and, during one shift, only they were wearing regular disposable masks. The researcher witnessed two nurses talking with each other. One of them said: “Did you hear that one of the nurses died due to COVID-19?” The other nurse said while laughing: “oh, really? Then let’s disinfect our hands,” and the other nurse said: “No need for that; we won’t get the virus!”

### A compassionate nurse

Some nurses were famous for their sympathy in the ward. These nurses’ characteristics included proper communication with the patient and respectful behavior, and providing compassionate and professional care. The researcher observed them communicating well with the patients and their companions and kindly answering their questions. Another nurse patiently administered the medicines and said to one of the patients: “The medicine I injected for you was morphine, a strong narcotic pain reliever. Don’t worry. After a few minutes, your pain will subside.” One of the patients said: “Ms. X is very kind; she always behaves sympathetically; I get happy when she is in the ward.” Nurses were friendly and considerate of their colleagues. One of them said: “I understand my colleagues. If someone has a problem and can’t come to his/her shift, I change my shift with them. I may have a problem someday. I’d like to cooperate with my colleagues as much as possible.”

### A moonlighting nurse

During her field observations and talking with nurses, the researcher realized that some nurses worked simultaneously in two hospitals due to low salaries and economic problems. The personnel entitled them “moonlighting nurses.” One of the nurses said: “Ms. X is a moonlighting nurse; she is swamped.” Another nurse said: “I have to work in two hospitals at the same time because of my parents’ illnesses and the high treatment cost.”

### A drug bartender

Due to their excruciating pains, the patients requested frequent painkiller injections from the nurses, and during the shift, the nurse injected narcotic painkillers for one patient several times. One of the nurses said: “Patients repeatedly ask us to inject narcotics; after a while, their bodies become resistant, and the drug dose should be increased. It makes me feel I’m a bartender, but I don’t like”. Another nurse said, “Here, we take the role of drug distributor for some patients, and they won’t calm down until they get painkillers.”

## Discussion

This study provided a deep cultural insight into nursing care in the oncology ward, considering this ward’s specific culture and emphasizing the intertwined roles of nurses. Our study showed that oncology nurses played eleven interwoven roles voluntarily or involuntarily. According to the nature of nursing care in this ward, these roles are on a spectrum, with positive roles on one side and negative roles on the other. Taking the role of Robin Hood, nurses acted as the legendary hero Robin Hood and considered themselves saviors and protectors of patients. Although Robin Hood is known as an outlaw, his behavior can be justified by creating justice and helping the poor [21]. Economic sanctions have had a paralyzing effect on Iran’s healthcare sector. Among the victims of this sanction are cancer patients whose treatment has become inaccessible. This is a bitter experience since chemotherapy drugs are costly, and society’s middle and lower classes have limited access to these drugs [22]. Shahabi et al. state that in Iran, due to the existing sanctions, cancer patients face a serious medication shortage resulting from medication transportation, currency transfer problems, and a lack of funds [23]. It is believed that nurses’ unethical behavior in the role of Robin Hood, which was for the benefit of the patients under that conditions, was ethical.

As secretive nurses, they concealed three types of truth: concealing the diagnosis of the disease from the patient at their family’s request or inversely, concealing the nurse’s error from other personnel, and nursing managers being secretive about privileges given to the ward to other personnel. In this study, the nurses did not reveal the disease diagnosis to the patient at the request of their family. The general principle is to respect the patient’s autonomy. Despite the consensus on disclosing the diagnosis, non-disclosure is still a common practice in the Middle East, where culture is focused on family and community values, and this cultural context complicates the issue [24]. Disclosure of the cancer diagnosis to the patient is still commonplace in these areas [25]. In line with the results of the present study, in a study conducted in Bahrain, 50% of the medical staff members refrained from disclosing the disease diagnosis to the patient at the request of the family members [24]. In this study, since the family had an accurate understanding of their patient’s mental capacity, they requested the nurse not to reveal the diagnosis due to the possibility of the patient’s disappointment and low morale. Considering the patient’s condition, the nurse decided to conceal the diagnosis. Besides, some patients were unwilling to disclose their diagnosis to their families.

Concealing an error committed by the nurse from others was another type of concealment due to fear of blame, error reporting, humiliation, punishment, and stigmatization by other personnel. Nurses are required to consider patient protection dealing with an error; however, some factors lead nurses to protect themselves rather than the patient since the dominant culture of hospitals necessitates concealing errors. Another type of secretive behavior was the concealment of privileges and items donated to the ward by nurse managers from other nurses. Insincere management leads to the nurses’ resentment and pessimism toward the organization [26]. Managers’ such behavior in the ward is an instance of toxic behavior and leadership that is, unfortunately, becoming frequent in nursing [27]. In order to maintain respect for the patient’s independence, providing clarifications on the disease diagnosis for nurses is essential. Moreover, to reduce error concealment, an encouraging culture should be created in the hospital; as a result, nurses announce their committed errors courageously. In addition, nursing managers are required to become aware of concealment in the hospital as a toxic behavior to apply more effective management.

Negligence was some nurses’ characteristic manifested in various dimensions of their professional duties, including the drug therapy process, psychological care of the patient, delegating tasks to others, monitoring vital signs, changing dressings, instructions and regulations, patient treatment process, and patient training. These nurses provided care as routine tasks, sometimes incompletely. It is believed that missed nursing care occurred in this ward. Likewise, in other studies, nurses’ lack of caring behavior was reported [15]. The concept of missed nursing care in oncology wards is of particular importance [4]. In studies, this issue has been addressed from various dimensions. In line with the results of the present study, Griffiths et al., in a review, reported that 75% of nurses or more missed some care [28]. In another study conducted in Turkey, one of the themes extracted was the lack of caring behavior in some oncology nurses; they only performed routine and daily tasks [4]. Furthermore, consistent with the results of our study, a study carried out in Poland stated that the most missed nursing care included recognizing and evaluating the patient’s condition, psychological support for the patient and their family, and punctuality in performing tasks [29]. Another type of nurse negligence was delegating duties to patients’ companions or assistant nurses. In another study conducted in Iran, nurses assigned some tasks to patients’ companions or assistant nurses [4]. According to the results of the present and other studies, it can be stated that negligence is a common practice among oncology nurses, which is influenced by the culture of this ward. Since nurses’ negligence might threaten the quality of patient care, it should be taken into account by supervisors.

Snitching was manifested as exaggerating colleagues’ errors, disparaging them to the managers and other colleagues, attempting to undermine colleagues’ positions, and transferring them to another ward. This role can be an important challenge in the nursing profession since it contradicts the ethical nature of this career. Snitching, considered as stabbing, is an anti-social behavior [30] and a subset of horizontal violence in the ward. Horizontal violence is a frequent behavior and a critical challenge in the nursing profession [31], which is described as an enthusiasm killer in the workplace [30].According to a study conducted in England, many nurses complained about gossiping, which was a characteristic of most hospital environments where colleagues were used to criticizing and snitching [32].It can be stated that nurses, in the snitching role, face situations where they experience a break in relationships with their colleagues. As a result, the provision of quality care, which requires a cooperative spirit and team communication between nurses, does not occur optimally, and thus the patient is harmed.

Nurses’ other characteristic was the complainer. They mainly complained due to job dissatisfaction and working conditions. In Brazil, caring for cancer patients was associated with the medical team’s job dissatisfaction [33].The characteristics of the workplace affect nurses’ satisfaction [34].One of the reasons for nurses’ dissatisfaction with their profession in the ward was the increase in the ratio of patients compared to nurses. Similar results were also obtained in a review [35].Considering that nurses’ job dissatisfaction has been the focus of nursing researchers in studies as an organizational problem and can influence nurses’ clinical performance, nursing managers should take this negative role into consideration to provide job satisfaction for nurses.

The other role taken by nurses was an apathetic senior nurse. This role was manifested in senior nurses with a sense of fatigue, burnout, low value of the profession, less desire and yearning to perform duties, and freedom from the profession. It appeared that these behavioral characteristics in nurses were a manifestation of job burnout. In fact, the dominant culture in the oncology ward causes these wards to be considered as high-risk for nurses to suffer from the mentioned behavioral characteristics since oncology nurses often observe patients’ unrelieved pain, witness their suffering, experience constant and sometimes overwhelming emotional stress, and have to manage complex injuries with poor prognosis [36]. On the other hand, all the conditions mentioned in this and other studies can affect nurses’ physical and mental health, the quality of their professional life, and ultimately the quality of patient care and satisfaction. According to the researcher’s observations, no organizational psychologist was employed in the oncology ward; however, at least one psychologist is needed to exempt the unenthusiastic senior nurse from the aforementioned behavioral characteristics, resulting in high-quality patient care.

Another title specific to newly-graduated nurses was ’a stigmatized nurse.’ These stigmatized nurses had to admit being subordinates, remain silent in the face of injustice, tolerate other personnel’s inappropriate and unfriendly behavior, and be content with less than their rights. Studies have paid attention to the transition process of newly graduated nurses for whom transition experience is challenging [37]. In line with the results of the present study, newly graduated nurses’ emotional exhaustion, stress, inability to meet job demands, incapability to provide safe care, fear of the physician, fear of error, and lack of support have been mentioned [38]. However, these nurses’ working conditions can be improved by creating a supportive organizational culture, valuing them, and personnel’s and nursing managers’ unbiased behavior toward them.

The other finding was a brazen-bodied nurse. In other words, similar to the legendary hero, Esfandiar Ruin Tan, no harm or danger threatened their physical and mental health; therefore, they expected a strong and invulnerable body from themselves and others. Invulnerability implies that one has a mythical assumption of ​​physical and mental immunity against distress and harm. During this study, the COVID-19 pandemic put the country in the red rank based on the color-code list and proved that nurses were committed to their profession and patient care. However, their heroic actions in the current era should not lead to the false impression that nurses are invulnerable to COVID-19 and its related health risks [39]. Unfortunately, the false belief of being brazen-bodied existed in some ward nurses.

Compassion is recognized as the main component of care, and oncology nurses need to be compassionate when caring for oncology patients from diagnosis to the survival or end of the patient’s life [40]. In this study, the role of a compassionate nurse was in line with the description of compassion for nurses in other studies, in which sympathy was defined as kindness, attention to and understanding of the patient, recognizing patient needs, establishing proper communication with the patient, understanding the patient’s feelings, and empathizing with them [41]. These nurses were known as “kind nurses” among patients. Culture is one of the external factors that can facilitate compassion [41]. Considering the culture and value Iranian activists place on kindness and helping others, expecting nurses to accept this role was not far-fetched.

A moonlighting nurse was another role; nurses simultaneously worked in public and private hospitals due to low income, and they were known as ‘moonlighting nurses’ for the personnel. In other studies, nurses had a second job for financial reasons [42, 43]. Moreover, this study was conducted during the COVID-19 crisis, when we faced a severe shortage of nursing staff, which facilitated working two jobs for nurses. McDonald et al. likewise stated that approximately two-thirds of hospitals had staffing shortages during the COVID-19 pandemic, and as a result, they relied on moonlighting nurses [44]. Few studies have addressed the effect of nurses’ second jobs; however, it is generally considered objectionable and is a hidden element whose effect on care has not been identified [45]. Hospital managers should create conditions to improve nurses’ economic status so that they will not feel the necessity to have a second job since working two jobs decreases the quality of nursing care in oncology wards by imposing fatigue.

Another role of the nurses was drug bartender, a title in Iran given to a person who distributes drugs. Due to their excruciating pain, the patients frequently asked the nurses to inject narcotics; therefore, the nurses relieved their pain. Pain caused by cancer was a constant challenge for oncology nurses, and due to their unbearable pain, patients frequently asked nurses to inject narcotic painkillers; consequently, nurses considered themselves “narcotic bartenders” in the ward. Unalleviated pain in cancer patients leads to disturbed comfort, depression, social isolation, anxiety, desperation, and generally reduced quality of life. Uncontrolled pain even leads to suicide [46]. Pain is strongly influenced by the social and cultural context. Culture influences the way pain care is provided [47]. However, due to the prevailing culture in the oncology ward and the frequency of patients’ requests to receive narcotics, the nurses made an effort to relieve the patient’s pain, about which they were not pleased since they considered themselves narcotic bartenders.

Focusing on nurses’ roles regarding the specific culture of the oncology ward and using the focused ethnography method were among the strengths of the present study, the first study in this field conducted in Iran. However, due to a limited number of studies in this field, the authors lacked the chance to comprehensively compare the findings obtained from different dimensions, which can be considered one of the limitations of this study. Moreover, since the present study was conducted in only one oncology ward, the results should be cautiously generalized. It should also be noted that the informal interviews were conducted at the earliest possible time after the observations; however, they were sometimes postponed due to the ward’s busy schedule, which could affect nurses’ perceptions. To avoid it, the triangulation method was used for data collection.

## Conclusion

The results of this study added to the body of nursing knowledge and revealed nurses’ intertwined roles regarding the subculture of the oncology ward. Some roles, such as the compassionate nurse, facilitate the provision of quality patient care, whereas other characteristics, such as the complaining nurse, may be an obstacle to providing such care. Although changing the culture of the oncology ward is not an easy task, it is achievable. The results of this study can be provided to nursing managers; therefore, by being aware of nurses’ roles considering the specific subculture of the oncology ward, they can provide psycho-educational interventions to improve the mental health of reluctant and complaining nurses and ethics-based training for secretive, negligent, and snitching nurses to provide quality care to the patient. Through some support strategies, such as recruiting efficient nursing staff, it is possible to solve the problem regarding the disproportion between the number of patients and nurses in order to alleviate the dissatisfaction of complaining nurses. Furthermore, it is essential to reduce nurses’ secretive and snitching behaviors, which can be achieved by holding educational workshops for nurses on communication skills in the hospital. Besides, nursing managers can solve moonlighting nurses’ economic problems and improve their living conditions utilizing a supportive approach. Further research in this field is suggested according to the culture of the oncology ward in other countries.

## Data Availability

The data supporting the findings of this study are available on request from the corresponding author. The data are not publicly available due to privacy or. ethical restrictions.

## Footnotes

1 Esfandiar was a mythical prince of Iran who was washed in holy water when he was a newborn and became brazen-bodied.
